# Notes on Ocular Therapeutics

**Published:** 1886-06

**Authors:** F. Richardson Cross

**Affiliations:** Surgeon to the Bristol Eye Hospital; Ophthalmic Surgeon to the Bristol Royal Infirmary


### NOTES ON OCULAR THERAPEUTICS. By F. Richardson Cross, M.B. Lond., F.R.C.S.; Surgeon to the Bristol Eye Hospital; Ophthalmic Surgeon to the Bristol Royal Infirmary.

A Case of Poisoning by Duboisia.—A healthy lady, of about 50, was sent me by the late Mr. Michell Clarke, with headache and asthenopia. Not being satisfied with my examination and results, I directed her to put one of Savory and Moore's Duboisin discs into each eye before seeing me on the following afternoon. During the afternoon I received a message from Mr. Clarke, asking me to call on his patient, who had been very ill, but was better. At about five o'clock I saw her, and obtained the following particulars: At 12.30, Mrs. W. put a disc into each eye, and almost at once felt pain in them and headache. She sat down for a quarter of an hour, then walked to the dining-room, remaining perfectly still and quiet, complaining of giddiness in the head. She got to the sofa, was very tottering and weak, but perfectly conscious. At one o'clock, when dinner was on the table, she stayed on the sofa, but was willing that meat should be cut up for her, though she did not eat it, and she constantly asked for water. She then began talking indistinctly, her ideas being natural, but her speech muffled. Some tea was brought her, which she tasted, but could not hold the cup herself. She seemed as though faint, and her hands were very cold: said she felt as though she were dying, and consented to the doctor being sent for. When Mr. Clarke came, he rapidly found the case of the Duboisin discs, and applied appropriate remedies, but no strong drug as an antidote. She soon became quite delirious, very restless, excited, and garrulous, tossing about from one side of the sofa to the other, fancying herself a frog, and mimicking its movements, by trying to crouch and jump on the floor, picking up all kinds of fancied things. She said she had sewing to do, and went through the pantomime of threading a needle: this she did many times. She could not walk without support; but after swallowing a little coffee with great difficulty, she seemed in a measure to regain the strength of her limbs, and was able to move about the room in a feeble, tottering way, talking incessantly and incoherently the whole time. When I saw her, at five o'clock, she was fairly quiet, and indistinctly appreciated her condition, and that she was ill. She had consented to lie down, but at times wanted to get up and move about, and was still very excited; her pupils large and staring, her pulse weak, her skin fairly warm and moist. She drank some tea without any difficulty. At 6.30 she was quite herself again, though talking rather much, and every now and then throughout the evening losing the thread of what she was saying. And during the following day she was still weak and prostrate.

Injurious Effects of Cocaine.—Among the very large number of cases in which cocaine has been used in ophthalmic surgery, not a few have been recorded by good observers where unpleasant and even serious consequences have been attributed to its application. Desquamation of the corneal epithelium is probably frequently found; but much more definite changes result after operations on the cornea, under anæsthesia by this drug, such as vesicular and parenchymatous keratitis, which may leave scarring of the cornea, and permanently interfere with vision; even panophthalmitis after cataract extraction has been attributed to its use. Some of the reported disasters may be assumed to be directly dependent on the drug, applied with every precaution; some are due to its application in too strong solution, or in too liberal a manner; whilst in others it has probably done no direct mischief, but has acted as the medium of septic inoculation. The gelatine discs may possibly readily take up septic material, as cocaine appears to be largely hygroscopic, and in solution it readily decomposes with the presence of septic organisms. Our cocaine solutions have for a long time been rendered aseptic by sublimate or boric acid, as is the case in most ophthalmic clinics; and Galezowski has introduced into his discs a sufficient proportion of mercuric chloride. We have not traced any local trouble to the use of the drug, which we have used in 4 per cent. and 10 per cent. strength, but in a few cases slight conditions of constitutional trouble have seemed the result of its application; and after two cataract extractions, the patients were sleepless and very excitable throughout the night; two others suffered from very severe headache, and two or three have had diarrhœa. There have been attributed by various observers to its use apathy, very marked malaise, headache reaching an extreme degree, dyspnœa, weakness in walking, difficulty of speech, cramps and trembling in the extremities, sickness, loss of appetite, and even delirium. A case is published where three drops of a 2.5 per cent. solution produced severe prostration. Cocaine is likely, as its price is lowered, to come into very common use; but it is evidently necessary to have some care in its application, more particularly, as published cases would seem to show, in children, old people,and worn-out constitutions. Preparations used should be sufficiently fresh, and discs containing it should be kept dry. A 2 per cent. solution is a more rapid and more transient mydriatic than the atropia group; it is especially useful in ophthalmic examination of nystagmus, where it distinctly lessens the movements.

Atropine Conjunctivitis.—The December number of the *Annales D'Oculistique* contains a paper by M. Glorieux (Louvain), giving results of experiments with an absolutely pure solution of atropine on the circulation in the web of frogs and in the ear of rabbits. A ligature, preventing afferent as well as venous circulation, surrounded each hind leg at the tibio-tarsal joints. The left foot was immersed in water, and the right in a solution of atropine 1 per cent. Control experiments were also made. In 24 hours the ligatures were removed. Each web became marked by hyperæmia; the right was more definitely and more persistently affected. The microscope showed the atropised vessels to be more dilated, and to present a closer network, and to contain masses of leucocytes,—some circulating with the blood, others lining the walls of the veins,—but no instance of diapedesis was seen. The condition was an inflammatory hyperæmia, requiring four or five days in which gradually to subside; and a similar result followed exposure to a 1 per cent. solution for 18 hours. The opposite web much more rapidly recovered its normal condition, having passed through hyperæmic changes of far less degree. In order to show that the changes depended on the atropine, similar experiments were made by exposing the right web for a shorter time to a 2 per cent. solution. Unequivocal signs of inflammation resulted in 8 hours, the opposite foot being comparatively unaffected. By employing stronger solutions, increased inflammatory conditions were set up. After 6 hours' application to the ligatured foot of a 4 per cent. solution of atropine, an hour after the section of the ligature, white globules crowding the field of the microscope were seen. Next day the capillaries were absolutely blocked by the abundance of leucocytes; in many veins the circulation passed in the form of corkscrews, because of the great number of white corpuscles which lined their walls. But swelling or œdema of the member scarcely ever resulted. Friedlaender's observations on this absence of swelling with inflammatory conditions of the frog's web are thus supported. Exposure for an hour to a 5 per cent. solution of atropine caused profound inflammation, which lasted for five days; and this solution caused gangrene after three hours' mere contact. M. Glorieux next repeated his experiments on a warm-blooded animal—the rabbit. Following Cohnheim's procedure for producing inflammation as the result of anæmia, he tied the base of the ears tightly round a cork placed in the concha, and injected into the right ear, by means of Pravaz' syringe, slowly and with antiseptic precautions, from half a centigramme to two centigrammes of neutral sulphate of atropine dissolved in a gramme of water. The impunity with which rabbits eat belladonna is well known; and to kill these animals a gramme of atropine is required,—ten times the dose necessary to destroy a man,—the poison rapidly passing out of the system, mainly by the urine. The injection was retained, by means of the ligature, in the ear for three, four, or five hours. When the ligatures were removed, the two ears became intensely infected, but more so on the right side, where the atropine was; this ear showing divisions and subdivisions of vessels which were indiscernible in the left. Next day the left ear had usually assumed its normal appearance, but the right remained red and swollen and hot; but one of the four classical symptoms of inflammation was absent—pain. Pricking and pinching the inflamed ear on various occasions produced no effect. Often, the tip of the hanging ear became ulcerated by friction with the roughness of the soil,—an effect not to be wondered at when the anæsthetic action of atropine is remembered. In four rabbits, a centigramme of atropine was injected into the right ear, and the ligature left from two to five hours: distinct manifestations of inflammation showed themselves. Six other white rabbits showed redness and inflammatory swelling after the employment of half a centigramme of atropine, and the altered appearances lasted several days. M. Glorieux, in many varied experiments, has always obtained in the rabbit, by the employment of atropine, an inflammation varying in intensity with the dose employed. This persistent hyperæmia is strong evidence in favour of the paralysing action of atropine on the muscular coat of the bloodvessels. The mere ligature of the base of the ear, as shown by the controlling experiments made on the opposite side, and by Cohnheim's experiments (where, after ligature for eight, ten, or twelve hours, not the least inflammation sometimes resulted), was not the cause of the inflammation. Moreover, in order to produce the paralysing effect of the atropine on the muscular walls of the arterioles, it is necessary to cut it off for some time from the channels by elimination. Injections into the ear without previous ligature of its root produce so little effect, that in an hour it is difficult to say into which side has been introduced water or atropine solution. A rabbit which had received two centigrammes ate its food just as it had done on the previous day. Follicular conjunctivitis, the result of atropinisation, is, then, dependent on the physiological action of the drug on the arterial muscular coats; and such cases usually occur amongst patients with a feeble vascular tone, who may so far be said to show an idiosyncrasy to the drug. Erythema and eczema of the lids, and even chalazion, may thus result from the injudicious use of this vaso-dilating agent, however pure it may be. The remedy is cessation of the atropine, or else the combination with it of an astringent like zinc sulphate or lead acetate, which act by tending to contraction of the vascular walls. In a different category are the cases of irritation from a chemically impure atropine, as well as those due to the use of a septic solution. Salicylic acid or weak corrosive sublimate might with advantage be combined with atropine drops.

Phlyctenular Ophthalmia.—The connection of this affection of the eye with the strumous diathesis has been very clearly shown in several cases attending my clinic. A young man, aged 20, with several scars from strumous abscess, and one the result of lupus, came with a white phlycten half an inch in diameter on the outer segment of the corneo-scleral junction. The swelling was neither a pustule nor a vesicle, but a mass of lymphatic cells, or leucocytes, suggesting a strong homology of structure with an enlarged lymphatic gland which was present in the submaxillary region of the same side. Irritation with calomel insufflation was soon followed by absorption of the phlycten. Some episcleral staining continued for some time.

A strumous-looking girl of 16 presented herself with a large mass of lymphatic glands close to the angle of the jaw, and phlyctenular conjunctivitis of the same side. The gland in front of the tragus on the temporo-maxillary joint was enlarged. The history of the case showed that whilst the large glandular mass had existed several months, the eye had only become affected quite recently for the first time. A slight conjunctivitis resulted in a day or two in phlycten, which soon became exaggerated into markedly-swollen conjunctiva and episcleritis, suggesting overdistension of the connective tissue lymph spaces of the conjunctiva by stasis in the lymphatic circulation. In this case I considered, after careful watching for many weeks, and on several occasions afterwards, that the abnormal conjunctival condition followed after, and resulted from, inflammation and enlargement of the glandular mass, and that the circulation from the conjunctiva through the transverse facial and temporal lymphatics was obstructed at the enlarged glands. Treatment of the latter by iodide of potassium ointment reduced its size, and the eye improved without any direct local treatment, though calomel insufflation hastened the result on another occasion.

In many cases the phlyctenulæ are accompanied by

œdema of the conjunctiva, rather than by hyperæmia. The masses of lymphatic cells constituting phlycten, whether superficial or episcleral, are, in my experience, best treated by calomel irritation, whatever may be the vascular condition of the eye. At the same time extra treatment is applied according to the position of the lesion. I have found weak eserin drops of great benefit in the numerous cases among children where the little cell masses lie on the periphery of the cornea. When the cornea itself is affected, cocaine is of great service, with some antiseptic such as salicylic or boric acid. The constitutional condition, sometimes anæmic and sometimes plethoric, also requires appropriate treatment. Should the phlycten undergo degeneration, forming a pustule or an ulcer, the calomel should be discontinued, and, usually, sedative remedies should be ordered; but at this stage a weak "yellow" ointment is frequently of much benefit.

CORROSIVE SUBLIMATE.—A solution, in strength 1 to 3000 for the lids and skin, 1 to 5000 for the mucous membrane, has been most freely used in nearly all my operations during the last eight months with excellent results, and, so far as I can judge, without producing any opacity (as is the case with lead-salts) in the corneal tissues. Carbolic solution is used after alcohol for purifying all instruments.

I have but little faith in iodoform as an antiseptic for eye lesions.

---

